# *Fragon*: rapid high-resolution structure determination from ideal protein fragments

**DOI:** 10.1107/S2059798318002292

**Published:** 2018-03-02

**Authors:** Huw T. Jenkins

**Affiliations:** a York Structural Biology Laboratory, Department of Chemistry, University of York, Heslington, York YO10 5DD, England

**Keywords:** molecular replacement, density modification, protein fragments, *Fragon*

## Abstract

Correctly positioning ideal protein fragments by molecular replacement presents an attractive method for obtaining preliminary phases when no template structure for molecular replacement is available. This has been exploited in several existing pipelines. This paper presents a new pipeline, named *Fragon*, in which fragments (ideal α-helices or β-strands) are placed using *Phaser* and the phases calculated from these coordinates are then improved by the density-modification methods provided by *ACORN*. The reliable scoring algorithm provided by *ACORN* identifies success. In these cases, the resulting phases are usually of sufficient quality to enable automated model building of the entire structure. *Fragon* was evaluated against two test sets comprising mixed α/β folds and all-β folds at resolutions between 1.0 and 1.7 Å. Success rates of 61% for the mixed α/β test set and 30% for the all-β test set were achieved. In almost 70% of successful runs, fragment placement and density modification took less than 30 min on relatively modest four-core desktop computers. In all successful runs the best set of phases enabled automated model building with *ARP*/*wARP* to complete the structure.

## Abstract 2

A new pipeline to solve structures by molecular replacement with ideal protein fragments is described and benchmarked against two test sets of mixed α/β and all-β folds at relatively high resolution.

## 1. Introduction   

The molecular-replacement (MR) approach (Rossmann & Blow, 1962 ▸), in which experimentally derived structure-factor amplitudes are combined with phases generated from a partially correct model, is the most common method used to solve macromolecular crystal structures, accounting for almost 80% of the X-ray structures deposited in the Protein Data Bank (PDB) in the last five years. When the differences between the search model and the new structure are small, it is usually straightforward to place the model, and refinement quickly improves the phases. In challenging cases where the only template structures available are from distantly related homologues, more sophisticated tools are required. Molecular modelling (DiMaio *et al.*, 2011 ▸; Qian *et al.*, 2007 ▸; Wang *et al.*, 2016 ▸) may be able to modify the template to generate a better model or, in some cases, *de novo* predictions may be sufficiently accurate. The application of maximum-likelihood approaches in the molecular-replacement search algorithms provided by *Phaser* improves the chance of positioning even very partial models correctly (McCoy *et al.*, 2007 ▸; Read, 2001 ▸). Once a potential solution has been found, iterative cycles of phase improvement *via* density modification and automatic chain tracing (Cowtan, 2006 ▸; Sammito *et al.*, 2014 ▸; Terwilliger, 2003 ▸, 2008 ▸; Thorn & Sheldrick, 2013 ▸) have increased the chance of success. However, it is still often impossible to solve the structure using these techniques: either no reasonable template can be identified or it cannot be correctly placed, or the initial phases calculated from even a correctly placed template are too poor to guide model improvement.

As most proteins contain secondary-structure elements (α-helices and β-strands), standardized fragments with ideal secondary-structure geometry can provide alternative MR search models. These fragments are likely to be highly similar to some regions of the unknown structure, but only represent a tiny fraction of the structure. *Phaser* (McCoy *et al.*, 2007 ▸) can position such fragments correctly, but the signal-to-noise ratio is very low and it is difficult to distinguish correct placements from incorrect placements. The challenge of this approach is then to reliably identify which of the many phase sets calculated from the potential solutions are good enough to trigger successful current phase-improvement procedures and to generate an interpretable map.

Several pipelines have been developed to build upon the power of *Phaser* to position small fragments, such as *ARCIMBOLDO* (Rodríguez *et al.*, 2009 ▸), *AMPLE* (Bibby *et al.*, 2012 ▸) and *FRAP* (Shrestha & Zhang, 2015 ▸). *ARCIMBOLDO* and *AMPLE* in particular provide a variety of ways to select fragments and to assess MR success (Bibby *et al.*, 2013 ▸; Sammito *et al.*, 2013 ▸, 2015 ▸; Keegan *et al.*, 2015 ▸; Thomas *et al.*, 2015 ▸). Both use *SHELXE* (Sheldrick, 2002 ▸, 2010 ▸) to probe whether any of the placed fragments provide sufficient phase information to lead to a complete model through iteration of density modification and chain tracing to build more atoms into the fragment. The use of chain tracing enables atoms additional to those in the initial fragments to be used for the calculation of phases in subsequent cycles, and thus if these are correctly placed then chain tracing contributes to the phase improvement. In addition, as *SHELXE* reports the correlation coefficient (CC) between experimentally derived normalized structure-factor amplitudes (*E*_obs_) and those calculated from the trace (*E*_calc_), this allows progress to be monitored. When the resolution of the diffraction data extends to better than 2.5 Å, a value above 25% appears to be a reliable indication of success (Thorn & Sheldrick, 2013 ▸).

The *Fragon* pipeline uses the ‘dynamic density modification’ (DDM) method coded in the program *ACORN* (Foadi *et al.*, 2000 ▸; Yao *et al.*, 2005 ▸) to test whether phases calculated from a starting fragment can be improved to generate an interpretable map. This approach has been shown to be successful with atomic resolution data: the structure of triclinic lysozyme at 1.0 Å resolution was solved from initial phases calculated from a single ten-residue ideal polyalanine α-helix (Foadi *et al.*, 2000 ▸), and other novel structures at atomic resolution have been determined using this approach (Chavali *et al.*, 2005 ▸; Dubrava *et al.*, 2008 ▸).

The premise is that if the initial phases are not completely wrong (*i.e.* the fragments are correctly placed), then the maps calculated with these phases will show correct new atomic positions, albeit at a low level. Previous work indicated that this was possible provided that the measured data extend to sufficient resolution to give atomic peaks (Foadi *et al.*, 2000 ▸). In the DDM procedure, σ(ρ), the standard deviation of the map density, is first calculated and density modification then proceeds as follows. (i) Negative density is replaced by zero. (ii) Positive density is replaced by ρtanh{0.2[ρ/σ(ρ)]^3/2^}. (iii) The modified density is truncated to *kn*σ(ρ), where *k* is by default 3 and *n* is the lower of the cycle number and 5 (Foadi *et al.*, 2000 ▸). The minimum and maximum values for truncation are 0.1ρ_max_ and 0.8ρ_max_, respectively, where ρ_max_ is the maximum of the map. The truncation reduces the bias from the fragment, but is progressively decreased over the first few cycles of DDM so as not to remove density that appears outside the starting fragment (Yao *et al.*, 2006 ▸). The largest difference between DDM and density-modification methods used in other programs such as the ‘sphere of influence’ method employed in *SHELXE* (Sheldrick, 2002 ▸) is that in DDM the map is modified solely according to the ratio ρ/σ(ρ), *i.e.* the modification applied to the density at any grid point in each cycle is not determined by whether the grid point is assigned to the protein or solvent region (Yao, 2002 ▸), and therefore the performance of density modification cannot be influenced by the value entered for the solvent content.

**Figure 1:**
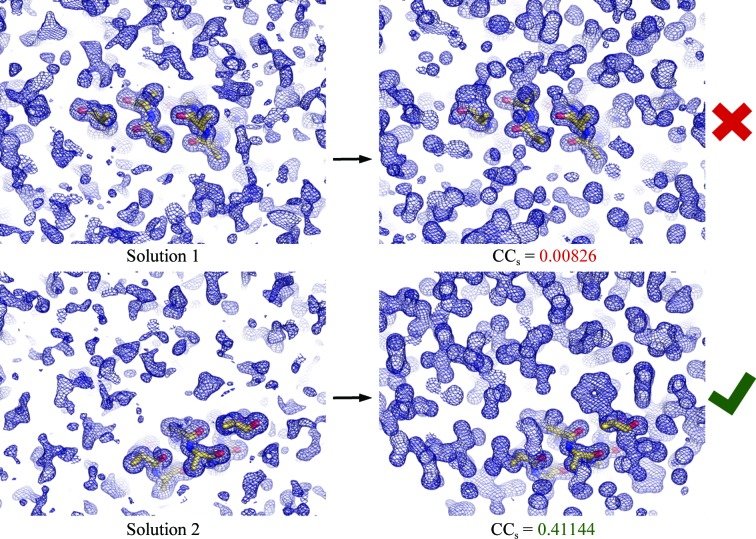
Phase improvement by density modification with *ACORN* illustrated for test case 1sxv (1.3 Å resolution) with phases calculated from a ten-residue ideal α-helix. Solutions are tested until the CC between the *E*_obs_ not used in the map calculation and their calculated values generated by back-transforming the modified map (CC_s_) indicates that phases from a solution have been sufficiently improved to enable automated model building.

*ACORN* modifies an atomic resolution *E*_obs_ map calculated using only the larger *E* values (in this work *E* > 0.8). It has been shown that also using ‘reflections’ beyond the measured resolution limit approximated as *E* = 1 enhances the map atomicity (Yao *et al.*, 2005 ▸). Previous work demonstrated that with measured data extending to 1.5 Å resolution, and starting phases calculated from fragments representing as little as 3.2% of the final model, these could be sufficiently improved to enable automated model building of the entire structure (Yao *et al.*, 2005 ▸). The current work extends this to starting with phases calculated from placed fragments with ideal secondary-structure geometry at similar resolutions. The basis of *Fragon* is that given data extending to sufficiently high resolution, the density-modification procedure within *ACORN* is powerful enough to reliably screen out incorrectly placed fragments (Fig. 1 ▸) and generate phases suitable for automated model building without the requirement for extensive cycles of chain tracing. Removing the requirement for chain tracing allows many more potential solutions to be tested with modest computing requirements. This makes it feasible to attempt challenging cases, where only one of hundreds of potential solutions is correct, on a desktop machine. Consideration of the ratio of the height of a peak at an atomic centre relative to the standard deviation of the electron-density map at 1.7 Å resolution with a phase error of 75° suggests that DDM is unlikely to be effective below this resolution (Yao *et al.*, 2005 ▸, 2006 ▸).

## 2. Methods   

### 2.1. Implementation   

**Figure 2:**
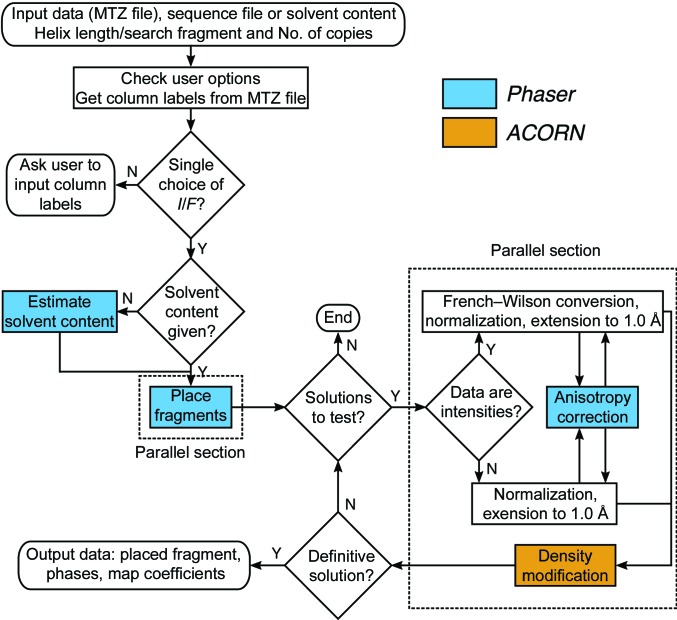
Flow diagram of the *Fragon* pipeline.

*Fragon* (Fig. 2 ▸) is essentially a Python wrapper with all the underlying crystallographic calculations performed by existing software. *Fragon* calls *Phaser* through its Python interface and *ACORN via* shell scripts. Manipulation of reflection data and coordinate files is handled by functionality provided by the *Clipper* (Cowtan, 2003 ▸; McNicholas *et al.*, 2018 ▸) and *cctbx* (Grosse-Kunstleve *et al.*, 2002 ▸) libraries.

Fragment location simply runs *Phaser* in its MR_AUTO mode with many options set to their default values. One important exception is that all solutions with clashing fragments are rejected (to speed up the run time). The default criteria for purging the list of solutions after the rotation and translation functions are used, but in searches for only one copy of a fragment the purge of solutions after rigid-body refinement is removed to output more potential solutions to test. In multi-copy searches this final purge governs the number of rotation functions performed in the subsequent fragment search, and so if the default cutoff [removal of solutions with log-likelihood gain (LLG) lower than 75% of the difference between the mean and top LLG] retains more than 100 partial solutions only the top 100 are kept.

### 2.2. Selecting test sets   

Previous work showed that only a tiny fraction of the structure was required to generate starting phases that could be improved by *ACORN* (Yao *et al.*, 2005 ▸), and this suggested that *Fragon* might be able to solve structures when only one or two small fragments were placed. This would be particularly powerful for determining structures where only a small part of the fold could be represented by an ideal α-helix. Previous approaches were able to produce success rates of over 80% for all-α test cases (Bibby *et al.*, 2012 ▸; Keegan *et al.*, 2015 ▸; Sammito *et al.*, 2015 ▸); therefore, in this work no all-α test cases were used and instead two test sets were generated, the first containing mixed α/β folds with limited α-helical content and the second containing all-β folds.

#### 2.2.1. Mixed α/β test set   

A test set of mixed α/β folds was selected from the PDB. The criteria applied were as follows. (i) Mixed α/β folds with a ratio of α:β content of less than or equal to 1. (ii) Data resolution between 1.0 and 1.7 Å. (iii) A single chain of 80–200 residues in the asymmetric unit. This meant that for the largest structures a model fragment of 7–10 residues would represent about 3–5% of the asymmetric unit. (iv) As the presence of heavy atoms facilitates density modification, structures containing elements heavier than chlorine were removed. (v) The test set was further filtered with *PISCES* (Wang & Dunbrack, 2003 ▸) to remove structures with detectable sequence identity. The final test set contained 103 structures (see Supporting Information).

#### 2.2.2. All-β test set   

MR with fragments of β-sheets [either extracted from structures in the PDB (Sammito *et al.*, 2013 ▸) or generated by the truncation of *ab initio* models (Bibby *et al.*, 2012 ▸; Keegan *et al.*, 2015 ▸)] is much more challenging than using ideal α-helical fragments, as the varied geometry of β-strands in β-sheets usually requires large libraries of β-sheets to be sampled in order to identify one with a similar r.m.s. deviation to a region of β-sheet in the target as that which an ideal α-helix has to many sections of α-helix in proteins. To test structure determination using idealized β-strands, a test set of all-β structures was selected from the PDB. The criteria applied were as follows. (i) No residues assigned as α-helical by *DSSP* (Kabsch & Sander, 1983 ▸). (ii) Data resolution between 1.0 and 1.7 Å. (iii) A single chain of 80–200 residues in the asymmetric unit. This test set was further filtered as described in §[Sec sec2.2.1]2.2.1 to leave 74 structures (see Supporting Information).

#### 2.2.3. Deposited data   

For both test sets the high-resolution limit reported in the PDB deposition was used to select and filter structures; however, for test cases where the deposited data extend to higher resolution (12 in total) all deposited data were used. For PDB entry 2pnd in the all-β test set the deposited data extend to 0.97 Å resolution (but are only 22.8% complete in the range 1.0–0.97 Å). No analysis was attempted to detect anisotropy in the data sets.

As *Phaser* now uses a log-likelihood-gain target based on intensities and their associated experimental error estimates (Read & McCoy, 2016 ▸), these were used when available in preference to structure-factor amplitudes.

### 2.3. Testing approach   

#### 2.3.1. Success criteria   

Successful runs were identified on the basis of the value of the CC between the smaller *E*_obs_ not used in the map calculation and their calculated values generated by back-transforming the modified map reported by *ACORN*, referred to as CC_s_. For all test cases in this work, CC_s_ was calculated for reflections with *E* values between 0.1 and 0.8. If the best CC_s_ was above 0.2 the run was deemed to be successful. In more marginal cases, where the best CC_s_ was between 0.09 and 0.2, if either the difference between the best and worse CC_s_ was greater than 75% of the best CC_s_ or the number of solutions was less than or equal to 10% of the maximum allowed (so as not to reject runs where there were multiple correct solutions and no incorrect solutions) the run was deemed to be successful. All successful runs were verified by using the improved phases from the *ACORN* run with the highest value of CC_s_ for automated model building with *ARP*/*wARP* (Langer *et al.*, 2008 ▸). For all successful runs except for those from PDB entry 4gu2, *ARP*/*wARP* was able to build a model with *R*_free_ below 0.3. For 4gu2 the best models after refinement with anisotropic ADPs had *R*_free_ values in the range 0.33–0.36, but since the deposited structure (1.35 Å resolution) and the rebuilt and re-refined model from *PDB_REDO* (Joosten *et al.*, 2014 ▸) have *R*_free_ values of 0.269 and 0.273, respectively, this case was also deemed successful.

#### 2.3.2. Mixed α/β folds with ideal α-helices   

Bias towards the known structure was avoided by making no attempt to tailor the search fragment. For each test case eight separate runs searching for one copy of an ideal (φ = −57.8°, ψ = −47°) polyalanine α-helix of between seven and 14 residues were performed. Up to 100 potential solutions for each helix length were tested by density modification with *ACORN*. For test cases where no run was deemed to be successful by the criteria defined in §[Sec sec2.3.1]2.3.1, a further eight runs searching for two copies of an ideal polyalanine α-helix of between seven and 14 residues were performed.

#### 2.3.3. All-β folds with ideal β-strands   

Ideal polyalanine β-strands comprising between three and five residues with identical φ/ψ angles of −120/+115, −125/+120, −130/+130, −135/+135 and −140/+135° were generated with *Coot* (Emsley *et al.*, 2010 ▸). The β-strands were arranged in pairs with parallel and antiparallel orientations and tilt angles between the β-strands of 0–30° in 5° increments. Ensembles (containing five models) of individual β-strands and pairs of β-strands were produced with *phaser.ensembler* and sorted so that the structure closest to the mean was the first model in the ensemble. For the 20 atomic (*d*_min_ ≤ 1.2 Å) resolution structures in the test set, searches for one copy of an ensemble of single β-strands with lengths of three, four and five residues were performed. If these were unsuccessful, and also for the remaining 54 test cases, searches for one copy of each ensemble of pairs of five-residue β-strands (14 ensembles in total) were performed. In these runs the pair of β-strands is refined by *Phaser* as a single rigid body and the model output is the first structure in the ensemble (as no *a priori* information about which model in the ensemble has the lowest r.m.s. deviation to the target is available). In an attempt to increase the success rate, the *Phaser* option to refine individual chains as separate rigid bodies can be employed to optionally further refine either each of the two β-strands as individual rigid bodies or split the strands about the central C^α^ atom and refine each half of the β-strand as a rigid body.

### 2.4. Use of the deposited model to identify correctly placed fragments   

In these nonblind test cases, it is possible to determine whether each *Phaser* solution is correctly located by using the model from the PDB as a reference. In order to perform this rapidly, the following procedure was developed. Firstly, the allowed origin shift that optimally superimposed (*F*_calc_, φ_calc_) maps from each placed fragment and the deposited structure was calculated with *RESOLVE* (Terwilliger, 2000 ▸). This offset was applied to the solution and this was then superimposed on the deposited structure with *CSYMMATCH*. A small *Clipper*-based utility was written to calculate the correlation coefficient between (*F*_calc_, φ_calc_) maps calculated from both the fragment and the deposited structure in the region encompassed by the fragment. To maximize the discrimination between correctly and incorrectly located fragments, only backbone atoms were included in the calculation, the ADPs of both the fragment and deposited structure were set to a constant value and only grid points containing density for the fragment were included in the calculation. To avoid confusion with more commonly quoted CCs between (*F*_obs_, φ_calc_) maps, this measure is termed the ‘placement score’. The use of (*F*_calc_, φ_calc_) maps eliminates the need for atom matching, which would be required to determine coordinate r.m.s. deviation between the search fragment and the deposited structure.

### 2.5. Benchmarking   

Benchmarks were performed on desktop computers with a single Intel Core i7-6700 (8 MB L3 cache, 3.4–4.0 GHz) or Core i7-4790 (8 MB L3 cache, 3.6–4.0 GHz) processor and 16 GB RAM running Scientific Linux release 7.3. Hyperthreading is enabled on these processors but a maximum of four simultaneous threads (*i.e.* one per physical core) were used.

## 3. Results   

### 3.1. Overall performance   

**Figure 3:**
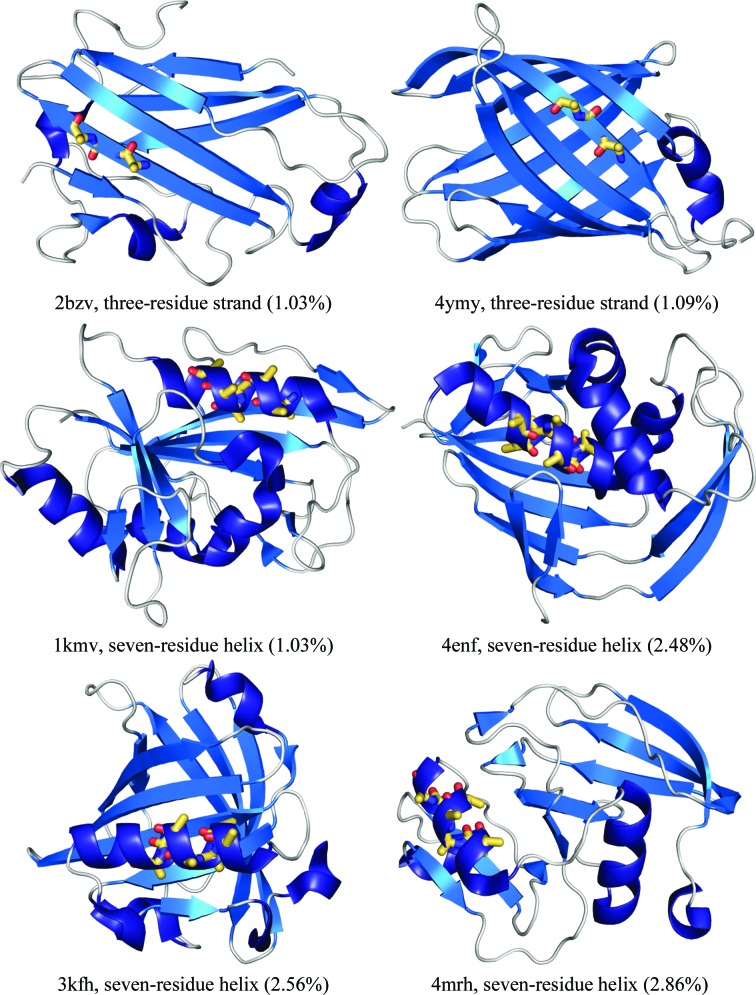
Selection of test cases solved by *Fragon* from fragments of ideal secondary structure accounting for under 3% of the total scattering. The fragment placed by *Phaser* is shown as yellow sticks and the deposited structure is shown as ribbons. In each case the PDB code of the test case, the size of the search fragment and the percentage of the scattering (as reported by *Phaser*) that this represents is shown.

As there was no overlap between the fragments used as search models in the tests performed in this work, *i.e.* the idealized β-strands were only used for the all-β test set and the ideal α-helices were only used for the mixed α/β test set, the overall performance of *Fragon* against the two test sets is described separately. The overall success rate (*i.e.* at least one run deemed to be successful using the criteria in §[Sec sec2.3.1]2.3.1) for *Fragon* against the mixed α/β test set of 103 structures was 61%. The overall success rate against the all-β test set of 74 structures was 30% (Table 1 ▸, Supplementary Figs. S1 and S2). Success was achieved with ideal fragments accounting for under 3% of the total scattering for 22 runs in the mixed α/β test set and 13 runs in the all-β test set: some examples are shown in Fig. 3 ▸. The success rate is correlated with resolution: in the mixed α/β test set it reached 89% for test cases at resolutions between 1.0 and 1.2 Å, decreasing to 68% for those with resolutions between 1.21 and 1.49 Å and further to 36% for those with resolutions between 1.5 and 1.7 Å. While the success rate of *Fragon* with ideal β-strands against the all-β test set is low, it is still encouraging as a small library of ideal strands (17 in total) was capable of solving 22 test cases. The options to further refine the β-strands placed as a pair as individual strands or to split each strand in half each resulted in success for two test cases. The success rate in the all-β test set was also better for structures at high resolution, with 65% of the test cases with resolutions between 1.0 and 1.2 Å solved. This includes six test cases solved by searching for one copy of an ensemble of single β-strands.

### 3.2. Fragment placement by *Phaser*   

**Figure 4:**
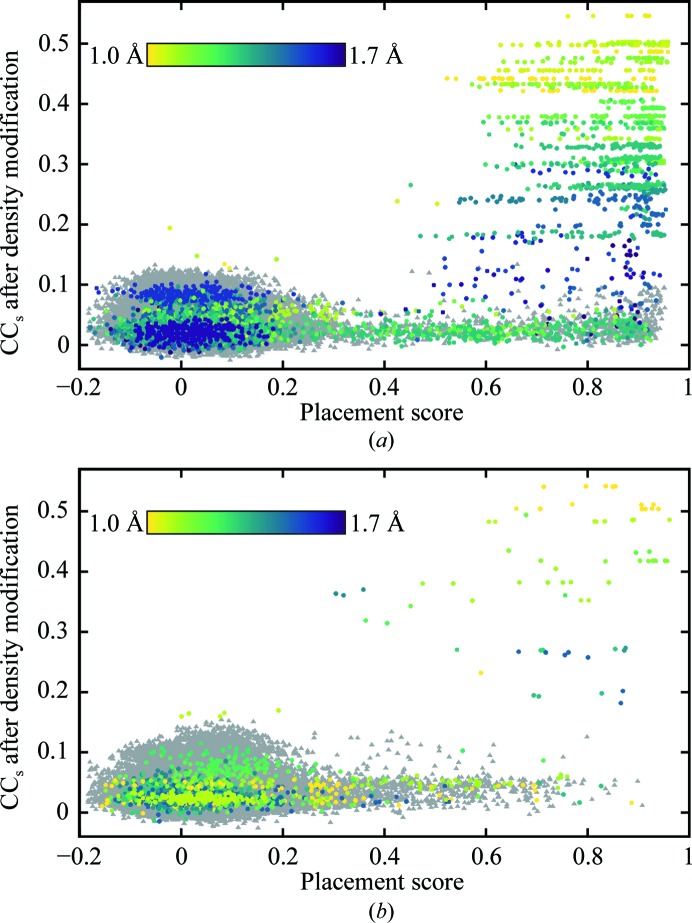
CC_s_ after *ACORN* density modification *versus* ‘placement score’ (§[Sec sec2.4]2.4). (*a*) Mixed α/β test set, (*b*) all-β test set. In both cases *Phaser* solutions from successful runs are indicated by circles coloured by resolution and *Phaser* solutions from unsuccessful runs are shown as grey triangles. For runs in which two ideal α-helices were placed the placement score for the second α-helix is shown, as these runs were only carried out when all runs with a single α-helix were unsuccessful.

The high redundancy of the testing performed here (multiple correctly placed fragments over all eight runs for some test cases; see Supporting Information), combined with the (*F*_calc_, φ_calc_) map correlation-based placement scoring (§[Sec sec2.4]2.4), enabled a huge number (nearly 170 000) of *Phaser* solutions (in the following the term ‘solution’ refers to a potential fragment placement) to be evaluated (Fig. 4 ▸). If a placement score of 0.3 (the edge of the cluster of low-scoring solutions in Figs. 4 ▸ *a* and 4 ▸ *b*) is taken as the lower bound for a correctly placed fragment, 2505 (2.85%) of the solutions in the mixed α/β test set and 850 (1.04%) of the solutions in the all-β test set are above this threshold. Such a small fraction of correctly placed fragments illustrates the scale of the challenge. Moreover, in the case of ideal α-helices there are often multiple correctly placed solutions from a single run, corresponding to one-residue shifts of the short search fragment along a helix in the target structure. It is important to note that while low values (*i.e.* less than 0.3) of the placement score clearly indicate an incorrectly placed solution and placement scores in the range 0.8–1.0 indicate very accurately located fragments, intermediate scores are harder to interpret. For example, the placement score does not differentiate solutions where some atoms are very accurately placed but some extend into solvent from those in which all atoms are somewhat inaccurately placed. The phases calculated for the former situation are likely to be more correct than the latter.

### 3.3. Analysis of eLLG for runs   

**Figure 5:**
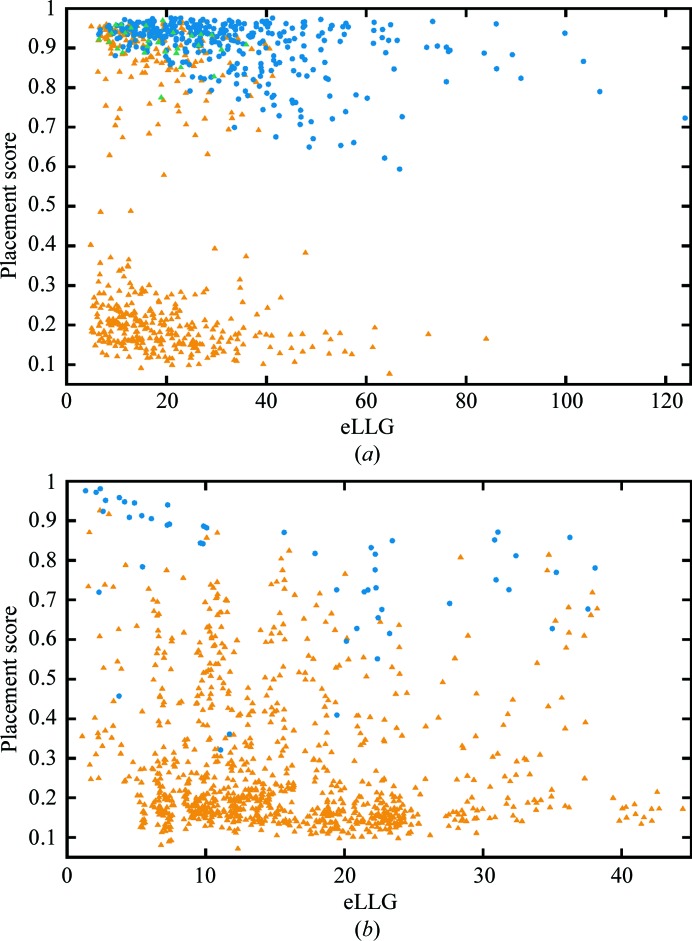
Placement score of the best-placed solution against eLLG for searches performed with one copy of an ideal α-helix or one copy of an ensemble of ideal β-strands or pairs of ideal β-strands. (*a*) Runs searching for one copy of an ideal α-helix in the mixed α/β test set (824 runs). Successful runs are plotted as filled blue circles, unsuccessful runs as orange triangles and unsuccessful runs for which runs searching for two copies of the same α-helix were successful as green triangles. (*b*) Runs searching for one copy of an ensemble of ideal β-strands or an ensemble of pairs of ideal β-­strands in the all-β test set (1012 runs). Successful runs are plotted as filled blue circles and unsuccessful runs as orange triangles.

Before an MR calculation is performed, the expected value of the LLG for a correctly placed model can be estimated. The eLLG is the total expected LLG summed over all reflections (McCoy *et al.*, 2017 ▸). The eLLG values *versus* the best placement score (§[Sec sec2.4]2.4) for the 824 runs in the mixed α/β test set are shown in Fig. 5 ▸(*a*). As expected for runs with low eLLG values, in many cases none of the *Phaser* solutions are correctly placed (placement scores clustered around 0.2), but as the eLLG increases an increasing proportion of runs contain (at least) one correctly placed solution. The proportion of successful runs increases with the eLLG, which is as expected as the eLLG increases with the fraction of scattering accounted for by the search model and the number of reflections (McCoy *et al.*, 2017 ▸). The 28 unsuccessful runs in the one-helix searches where searching for two copies of the helix was successful have eLLG values towards the lower end of the range. The same plot for the 1012 runs in the all-β test set (Fig. 5 ▸ *b*) is less informative. This is mainly because for each test case the pairs of β-strands have similar eLLG values, but it is clear from Table 1 ▸ and the Supporting Information that for a successful test case only one or a few of the ensembles resulted in success. The range of eLLG values is much smaller than for the helices in the mixed α/β test set, as the eLLG increases quadratically with the fraction of scattering (McCoy *et al.*, 2017 ▸), and the largest fragment used in the all-β test set is ten residues, compared with 14 residues in the mixed α/β test set. Interestingly, Fig. 5 ▸(*b*) shows that in four runs success came from fragments with low placement scores. In three of the four cases the low placement scores reflect that one of the ends of one or both β-strands in the fragment is placed into solvent, but in one case the strand lies across three strands in a β-sheet in the target structure. By chance some atoms are located at atomic positions in the true structure, so the mean phase error for the strongest *E* values (*E >* 1.6, 1999 reflections) is 80.8° and with data extending to 1.2 Å resolution *ACORN* was able to improve the initial phases calculated from this rather inaccurately placed model.

### 3.4. Identification of correctly placed fragments by density modification with *ACORN*   

Fig. 4 ▸ illustrates that the CC between the smaller *E*_obs_ not used in the map calculation and *E*_calc_ from the density-modified map (CC_s_) can reliably identify correctly located fragments. As would be expected, the discriminatory power of CC_s_ increases with higher resolution and, therefore, fewer of the *E*_obs_ used in map calculation approximated by *E* = 1. Figs. 4 ▸(*a*) and 4 ▸(*b*) reveal that there are cases where density modification fails to improve the phases from accurately placed fragments. In cases where these fragments are from runs that were ultimately successful, this has no impact on the overall success rate. However, there are fragments with high placement scores in runs that were unsuccessful, suggesting that further tuning of the parameters for density modification or placement of additional fragments may lead to increased success rates. Comparison of Figs. 4 ▸(*a*) and 4 ▸(*b*) suggests that the low success rate in the all-β test-set result is owing to searches with β-strands resulting in many fewer correctly placed fragments than searches with ideal α-helices in the mixed α/β test set.

### 3.5. Analysis of unsuccessful test cases   

All runs were unsuccessful for 40 test cases from the mixed α/β test set. Of these, in 20 cases the best placement score from all eight runs searching for one helix was in the range 0.15–0.36, indicating that no solution contained a correctly placed fragment. As up to 100 solutions were tested in the one-helix runs and the number of partial solutions kept in the searches for two copies of an ideal helix was limited to 100, for these 20 test cases two-helix searches would not be able to successfully place two copies of the search fragment. For the remaining 20 test cases the best placement score from all eight runs searching for one helix was in the range 0.62–0.96. Of these, six of the test cases only contained one α-helix, so ideal helices with lengths of 7–14 residues could only represent one part of the structure. Accordingly, in the runs searching for two helices for these test cases, no runs produced any solution in which both helices had a placement score of >0.3 in five test cases. The exception was PDB entry 1y9l, where the run searching for two copies of an eight-residue ideal α-helix produced one solution in which the helices were arranged with both fragments corresponding to parts of the 18-residue α-helix in this structure (placement scores of 0.88 and 0.59 for the first and second helix, respectively). For nine of the remaining 14 test cases none of the runs searching for two helices produced any solution in which both helices had a placement score of >0.3. Therefore, in only six of the 40 unsuccessful test cases were two copies of a helix correctly placed but density modification with *ACORN* was unable to improve the phases.

Of the 52 unsuccessful test cases in the all-β test set, the distribution of the best placement score from all runs for each test case is less informative. For 23 test cases it is <0.30, for 20 it is in the range 0.31–0.59 and for the remaining nine it is in the range 0.62–0.8. For the 29 test cases with best placement score of >0.30 the eLLG ranges from 6.3 to 23.7 for the corresponding runs, indicating that *Phaser* is unlikely to find a solution. For 26 of these runs the LLG of the top solution ranges from 26.0 to 65.1, but for three runs the LLGs of the top solutions are 123.4, 134.1 and 281.4. However, for these three test cases (PDB entries 4ld1, 4rlc and 4gei), 308 of 564, 27 of 141 and 144 of 144 solutions have LLG > 120, indicating that in these cases a high LLG does not definitively identify a correct solution. As Figs. 4 ▸(*b*) and 5 ▸(*b*) illustrate that fragments with placement scores in the range 0.3–0.8 can lead to success, for many of the unsuccessful test cases in the all-β test set failure cannot be owing to *Phaser* failing to correctly place fragments, but instead this must be because the fragments do not match the corresponding region of β-sheet accurately enough in the target structure for *ACORN* to be able to improve the phases.

### 3.6. Timing   

**Figure 6:**
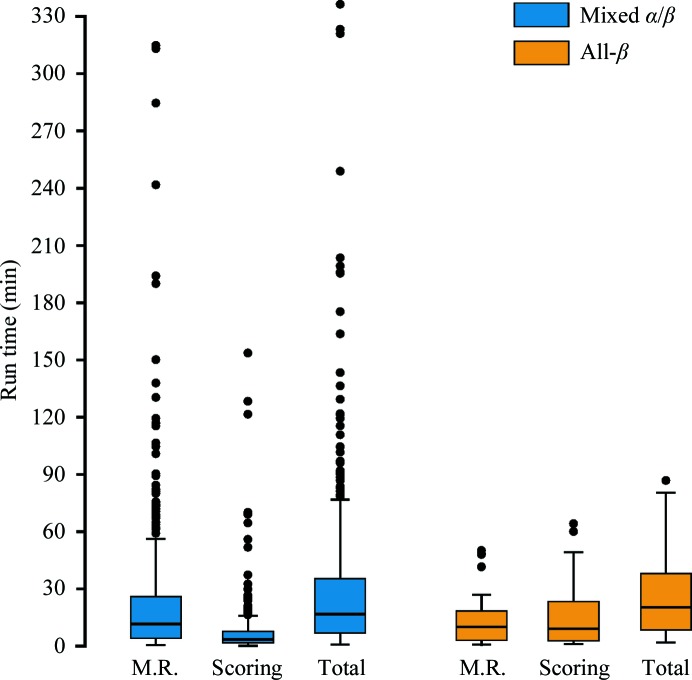
Box-and-whisker plots summarizing the run times for successful test cases. The whisker length is 1.5× the interquartile range. ‘M.R.’ is the time for fragment location with *Phaser*; ‘Scoring’ is the time for density modification with *ACORN*, which includes all time for reflection-file manipulation.

The highly redundant testing, together with the placement scores for all solutions (Figs. 4 ▸ *a* and 4 ▸ *b*), allowed the definition of criteria based on CC_s_ after density modification with *ACORN* that indicate that a definitive solution has been found and no further solutions should be tested. For atomic resolution data (*d*_min_ ≤ 1.2 Å) this is simply that CC_s_ is greater than 0.3. For data with resolutions between 1.2 and 1.7 Å this is once the difference between the highest and lowest CC_s_ for the solutions tested exceeds 0.15. As many solutions can be tested in parallel and running *ACORN* processes are not terminated once the first definitive solution has been identified, several definitive solutions may be produced before the run finishes. Applying these criteria to the 382 successful runs in the mixed α/β test set and 51 successful runs in the all-β test set allowed evaluation of the run times to be performed (Fig. 6 ▸). These benchmarks were performed on reasonably low-specification desktop hardware (§[Sec sec2.5]2.5). As no attempt was made to modify parameters based on the results of previous testing, the results are identical to those presented in Table 1 ▸.

#### 3.6.1. Fragment location with *Phaser*   

Fig. 6 ▸ illustrates how the time taken to place the fragment(s) dominates the run time in many runs and shows the greatest variability. This is not unexpected, as the MR_AUTO mode of *Phaser* has been carefully optimized so that the signal in the rotation function determines how many potential solutions are tested in the translation function. When the signal is low this can result in a long run time in which thousands of potential solutions are tested. However, if the translation function results in high-scoring solutions the many low-scoring potential solutions are discarded. For ten of the 15 successful runs in the mixed α/β test set with *Phaser* run times over 90 min this is the case and thus the translation function dominates the *Phaser* run time. All of these runs were from test cases in one of the 11 pairs of enantiomorphic space groups for which two translation functions are required to test both possibilities. Limiting the number of rotations tested in the translation function would speed up the run time, but since in two of the ten cases the highest scoring rotation was over 1000 places down the list this would have to be balanced against the risk of missing solutions. These ten runs are from four test cases and for each of these there were runs searching with alternative-length ideal α-helices in which the shortest *Phaser* run time was between 2.9 and 25% of that of the longest. In the other five runs with *Phaser* run times over 90 min the translation function failed to produce high-scoring solutions, and in two of these runs the rigid-body refinement dominates the run time (for the other three the translation function still required more time than the rigid-body refinement). It should be noted that in unsuccessful runs thousands of low-scoring potential solutions are retained throughout the run and the consecutive rigid-body refinement of these solutions accounts for most of the run time.

#### 3.6.2. Density modification with *ACORN*   

The median time for density modification with *ACORN* to identify a definitive solution in the mixed α/β test set was 3.48 min. The outliers with times longer than 40 min predominately reflect runs in which either the relatively conservative criteria for early termination were not triggered (three runs for test case 4xh7) or around 50% of the solutions were tested before the first correct placement was found. The only exception is for PDB entry 1sxv, where density modification with *ACORN* was particularly slow, requiring 50 min to test nine solutions. In the all-β test set the median time for density modification with *ACORN* to identify a definitive solution was 9.2 min and the two outlier times in Fig. 6 ▸ correspond to runs in which the first correct solution was numbers 67 and 92, respectively (shortest to longest time).

#### 3.6.3. Overall run times   

The shortest time required for density modification with *ACORN* in the mixed α/β test set was under 10 s and in 43 runs the time required was under 1 min. When the time for fragment location with *Phaser* was also very short this results in extremely short overall run times, with the fastest being under 40 s to solve the 1.15 Å resolution structure of monellin (PDB entry 2o9u; Hobbs *et al.*, 2007 ▸) with a single seven-residue α-helix. Of the 382 successful runs in the mixed α/β test set 55 took less than 5 min, 141 took fewer than 10 min and 265 (69%) took less than 30 min. The run times for the 51 successful runs in the all-β test set ranged from under 2 min to nearly 1.5 h; however, all but five runs took under 1 h and 34 runs (67%) finished in less than 30 min.

## 4. Discussion   

It is clear that when sufficiently high-resolution data are available, the placement of one or two secondary-structure elements such as an ideal α-helix or a β-strand followed by improvement of the phases calculated from the placed fragment by density modification can result in phases of sufficient quality to enable automatic model building to complete the structure. The challenge lies in testing a sufficient number of potential solutions to identify one that is correctly placed without relying on massive computational resources.

*Fragon* was implemented to address this challenge by enabling the rapid testing of potential solutions. The run time for successful *Fragon* runs is primarily governed by how easily *Phaser* is able to place the fragment, as this affects both the time for fragment location and how far down the solution list the first correct solution lies. For many of the test cases presented here *Fragon* requires less than 10 min on a relatively modest four-core desktop computer to solve the structure. Moreover, using the same hardware nearly 70% of the successful runs are finished in under 30 min.

This speed does not come at the expense of performance: the overall success rates for the mixed α/β test cases with ideal α-helices was 61%. An alternative approach is used in *ARCIMBOLDO_LITE* (Sammito *et al.*, 2015 ▸), where an improved ranking and filtering of potential solutions enables vastly fewer potential solutions to be tested than in the original *ARCIMBOLDO* (Rodríguez *et al.*, 2009 ▸) approach and thus, for easier target structures, the computational demands required for success to be vastly reduced. The 30% success rate for the all-β test set is encouraging as the same set of ensembles of β-strands (17 in total) was able to solve 22 structures, suggesting that the use of large libraries of β-sheets extracted from the PDB, as employed in *ARCIMBOLDO_BORGES* (Sammito *et al.*, 2013 ▸), is not always required. Comparison is difficult because the fragments used in *Fragon* represent idealized secondary-structure elements, *i.e.* the atomic positions are not derived from any template structure in the PDB and so the same fragments can be used for all test cases.

**Figure 7:**
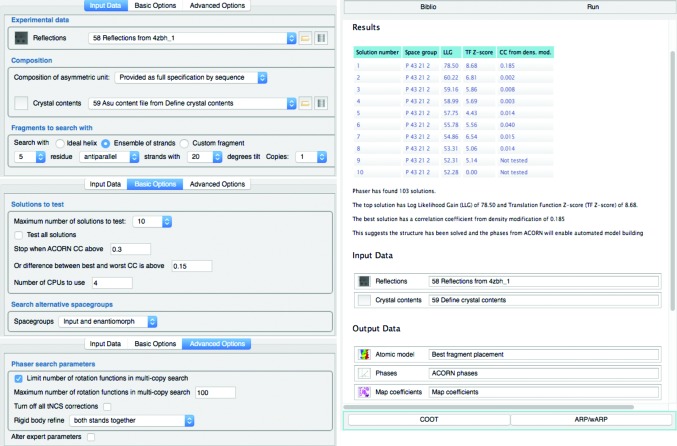
Solution of 4zbh using ideal β-strands with *Fragon* in *CCP*4*i*2. The input parameters are shown on the left and the report and output data on the right.

Powerful automated systems capable of generating vast numbers of results require well designed interfaces to cater to the needs of users with varying levels of expertise. In order to guide the user in choosing sensible options and most importantly to clearly present the results from hundreds of potential solutions, an interface to *Fragon* has been added to the *CCP*4*i*2 graphical user interface (Potterton *et al.*, 2018 ▸). Fig. 7 ▸ illustrates the use of this interface to solve the structure of the soluble domain of FlaF (PDB entry 4zbh; Banerjee *et al.*, 2015 ▸) from ideal β-strands. The structure of this 146-residue all-β fold was originally solved at 1.5 Å resolution by single isomorphous replacement with anomalous scattering from a platinum derivative. The user only needs to provide the reflection data and expected composition of the asymmetric unit, either explicitly by sequence or as an estimate of the solvent content. The interface allows easy selection of helix length or ensembles of β-strands and the detailed documentation helps to inform on a suitable choice of search fragment. The results are presented in a table (Fig. 7 ▸) and phases from *ACORN* for the best solution suitable for subsequent automated model-building pipelines and map co­efficients for viewing in *Coot* (Emsley *et al.*, 2010 ▸) are provided.

## 5. Availability   

*Fragon* will be submitted for distribution with the *CCP*4 suite and currently runs on Linux and MacOS operating systems. A graphical interface within *CCP*4*i*2 (Fig. 7 ▸) is available. The default parameters are those used for the benchmarks in this study.

## Supplementary Material

Supplementary Figures S1 and S2.. DOI: 10.1107/S2059798318002292/ba5279sup1.pdf

Detailed results for the performance of Fragon against both test sets.. DOI: 10.1107/S2059798318002292/ba5279sup2.xlsx

## Figures and Tables

**Table 1:** Performance of *Fragon* against two test sets

Test set	Resolution range (Å)	No. of structures	Search fragment	Copies	Runs	Solutions	Successful runs	No. solved
Mixed α/β	1.00–1.20	27	α-Helix	1	216	10603	168	24
	1.21–1.49	37			296	20675	138	22
	1.50–1.70	39			312	23335	48	9
	1.00–1.20	3†		2	24	2269	0	0
	1.21–1.49	15†			120	9993	12	3
	1.50–1.70	30†			240	21123	16	5
Total		103			1208	87998	382	63
All-β	0.97‡–1.20	20	β-Strand	1	60	4781	15	6
	1.00–1.20	14§	Two β-strands	1	196	16213	17	7
	1.21–1.49	28			392	31946	14	7
	1.50–1.70	26			364	28760	5	2
Total		74			1012	81700	51	22

† Test cases that were solved by searching for one copy of an ideal α-helix were not tested.

‡ One test case has a reported resolution of 1.0 Å, but the deposited data extend to 0.97 Å.

§ Test cases that were solved by searching with a single β-strand were not tested.
