# Supplementary material for: Fragon: rapid high-resolution structure determination from ideal protein fragments
Source: Acta Crystallogr D Struct Biol. 2018 Mar 2;74(Pt 3):205–14. doi: 10.1107/S2059798318002292 (PMC5947761; doi:10.1107/S2059798318002292)
Supplement: Supplementary file 1 [file d-74-00205-sup1.pdf]

STRUCTURAL BIOLOGY

Volume 74 (2018)

Supporting information for article:

Fragon: rapid high-resolution structure determination from ideal protein fragments

Huw T. Jenkins

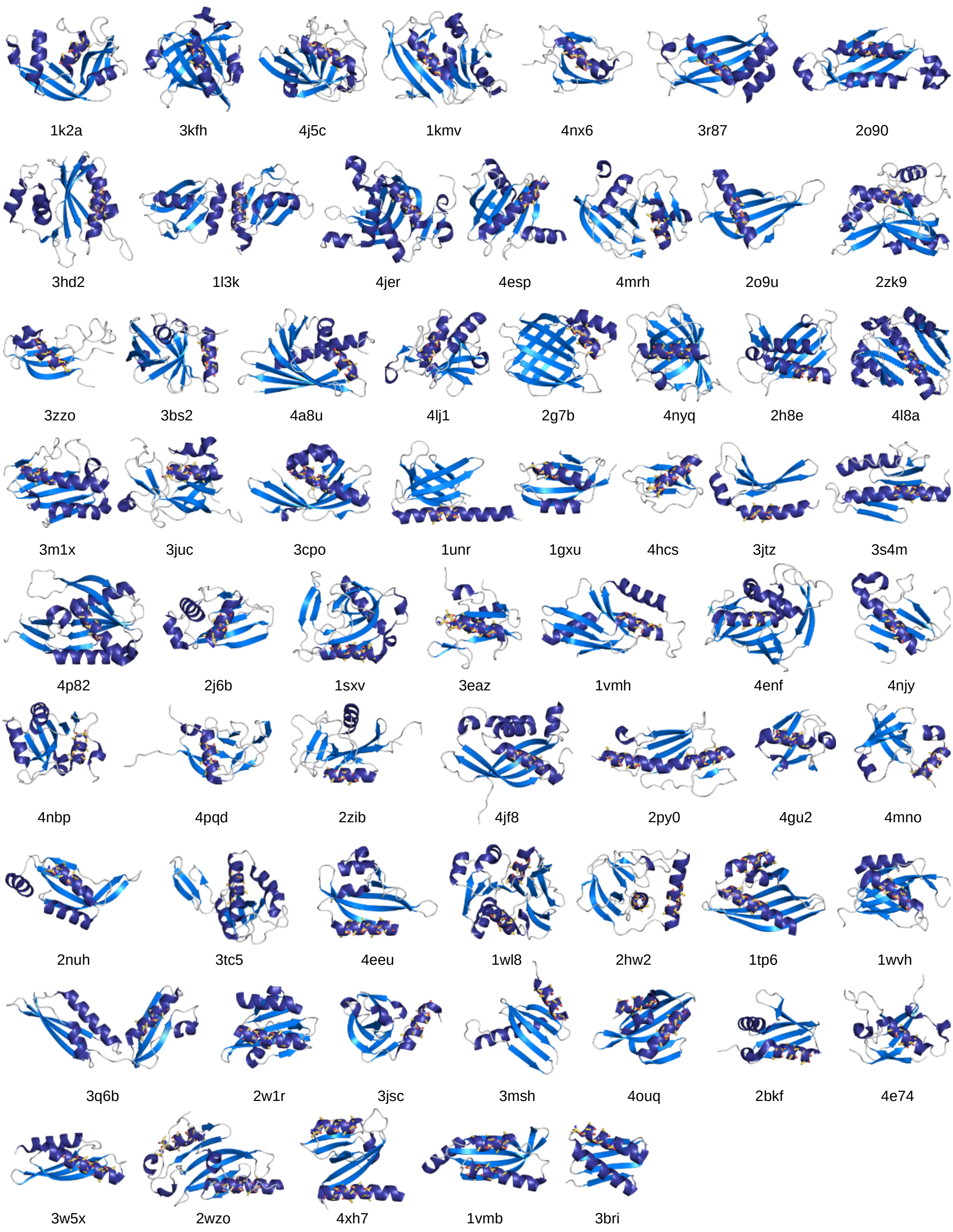

Figure S1 Test cases in the mixed α/β test set solved by *Fragon*. The fragment placed by *Phaser* is shown as yellow sticks.

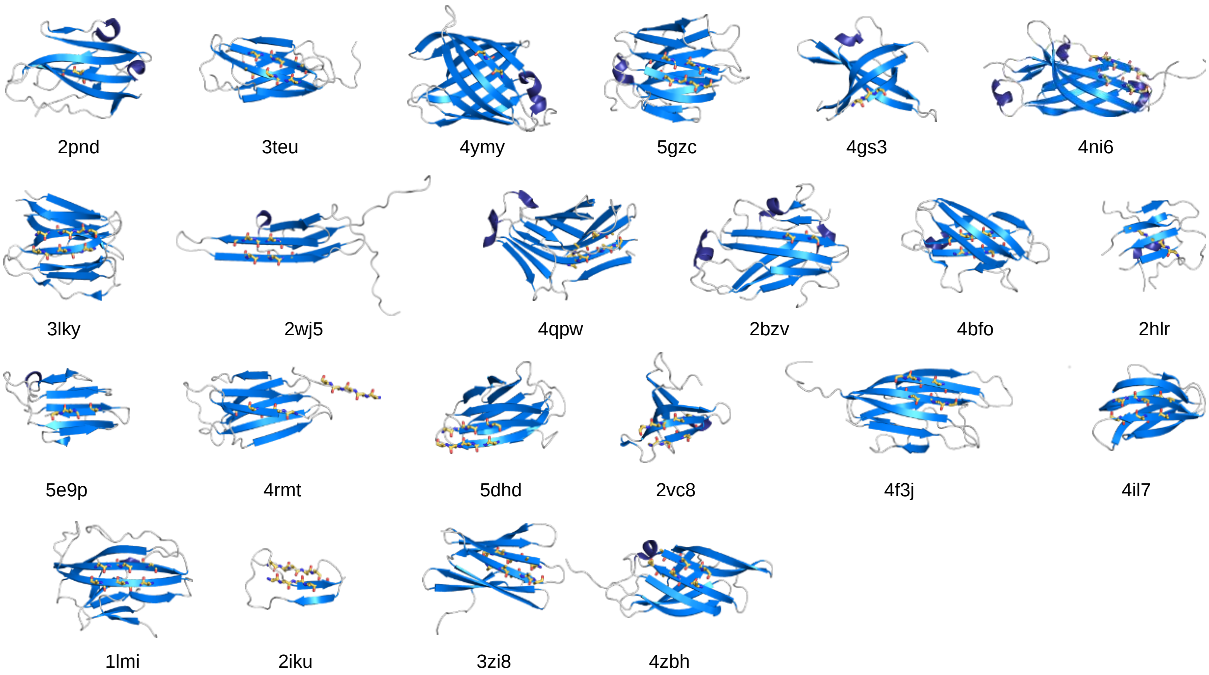

Figure S2 Test cases in the all-β test set solved by *Fragon*. The fragment placed by *Phaser* is shown as yellow sticks.
